# Supplementary material for: Molecular saturation determines distinct plasmonic enhancement scenarios for two-photon absorption signal
Source: Sci Rep. 2025 Jan 31;15:3956. doi: 10.1038/s41598-025-87198-y (PMC11785770; doi:10.1038/s41598-025-87198-y)
Supplement: Supplementary file 1 — Supplementary Information. [file 41598_2025_87198_MOESM1_ESM.pdf]

Supplementary Information for

Molecular Saturation Determines Distinct Plasmonic Enhancement Scenarios for Two-Photon Absorption Signal

Saeid Izadshenas Jahromi* and Karolina Słowik

Institute of Physics, Faculty of Physics, Astronomy, and Informatics, Nicolaus Copernicus University in Toruń, ul. Grudziądzka 5, 87-100, Toruń, Poland

1 Effective two-level description

Here, we derive the free-space form of the two-photon coupling strength in Eq. (2).

We consider a model molecule composed of ground and excited levels $|g\rangle, |e\rangle$ and a group of virtual levels $\{|i\rangle\}$ of energies $\omega_{g,e,\{i\}}$, respectively, and we assume $\omega_g < \omega_i < \omega_e$. The Bohr frequencies are $\omega_{mn} = \omega_m - \omega_n$ with $m, n \in \{g, e, \{i\}\}$. The free Hamiltonian is

$$H_0 = \hbar \sum_{j=g,e,\{i\}} \omega_j |j\rangle\langle j|, \tag{S1}$$

where the set labeled with $\{i\}$ includes all virtual states.

The system is subject to a monochromatic illumination with a frequency $\omega_l \approx \frac{1}{2}\omega_{eg}$ near the two-photon resonance. The interaction Hamiltonian is

$$H_{\text{int}} = \sum_i \hbar \left[\left(\Omega_{ig}^{(1)} |g\rangle\langle i| e^{i\omega_l t} + \Omega_{ig}^{(1)\star} |i\rangle\langle g| e^{-i\omega_l t} \right) + \left(\Omega_{ei}^{(1)} |i\rangle\langle e| e^{i\omega_l t} + \Omega_{ei}^{(1)\star} |e\rangle\langle i| e^{-i\omega_l t} \right) \right], \tag{S2}$$

where $\hbar\Omega_{pq}^{(1)} = \mathbf{E}_0(\omega_l, \mathbf{r}_m) \cdot \mathbf{d}_{pq}$ is the coupling strength of the free-space field with the amplitude $\mathbf{E}_0(\omega_l)$ evaluated at the molecular position with the molecular transition described with the dipole moment $\mathbf{d}_{pq}$. We have applied the electric-dipole and the rotating-wave approximations.

The intermediate levels are not, in general, spectrally positioned in the middle of the energy gap between the ground and excited states so that the single-photon resonance condition is generally not met. Here, we assume that the single-photon detuning between the field and the single-photon transitions $|g\rangle \leftrightarrow |i\rangle$, $|i\rangle \leftrightarrow |e\rangle$, is much larger than the two-photon detuning, but much smaller than the transition frequency scales:

$$|2\omega_l - \omega_{eg}| \ll |\omega_l - \omega_{ig}|, |\omega_l - \omega_{ei}| \ll \omega_l.$$

*Corresponding author: s.izadshenas@umk.pl

The next step to simplify the description is the adiabatic elimination of the intermediate state. The technique is a standard, well established tool which consists in reducing the dimensionality of the problem by eliminating a subsystem or a subset of states that is weakly and steadily occupied [1, 2, 3]. As a result, an effective Hamiltonian and corresponding equations of motion can be derived for a subset of relevant states that undergo nontrivial dynamics. In this case, the population of the middle state is typically small, and the state can be adiabatically eliminated. Below, we perform the elimination to find the relation between the single-photon and two-photon transition rates.

In the Schrödinger picture, the molecular state is a superposition $|\psi(t)\rangle = c_g(t)|g\rangle + c_e(t)|e\rangle + \sum_i c_i(t)|i\rangle$ with $c_g(0) = 1$ and $|c_g(t)|^2 + |c_e(t)|^2 + \sum_i |c_i(t)|^2 = 1$. The Schrödinger equation yields the following set of equations for the amplitudes:

$$\begin{aligned} i\dot{d}_g &= \sum_i \Omega_{ig}^{(1)} d_i \\ i\dot{d}_e &= (\omega_{eg} - 2\omega_l)d_e + \sum_i \Omega_{ei}^{(1)\star} d_i \\ i\dot{d}_i &= (\omega_{ig} - \omega_l)d_i + \Omega_{ig}^{(1)\star} d_g + \Omega_{ei}^{(1)} d_e. \end{aligned} \tag{S3}$$

where we have set $\omega_g = 0$ and substituted $c_e = d_e e^{-2i\omega_l t}$, $c_i = d_i e^{-i\omega_l t}$, $c_g \equiv d_g$ to approximately separate the free evolution.

Near the two-photon resonance, the virtual-level amplitudes are relatively small $|c_i(t)| \ll |c_g(t)|, |c_e(t)|$ and these states can be adiabatically eliminated from the dynamics

$$d_i = \frac{\Omega_{ig}^{(1)\star} d_g + \Omega_{ei}^{(1)} d_e}{\omega_l - \omega_{ig}}, \tag{S4}$$

which we plug back into the first pair of equations (S3). We find an effective two-level system description

$$i\dot{d}_g = \sum_i \frac{\left|\Omega_{ig}^{(1)}\right|^2}{\omega_l - \omega_{ig}} d_g + \sum_i \frac{\Omega_{ig}^{(1)}\Omega_{ei}^{(1)}}{\omega_l - \omega_{ig}} d_e, \tag{S5}$$

$$i\dot{d}_e = \left(\omega_{eg} - 2\omega_l + \sum_i \frac{\left|\Omega_{ei}^{(1)}\right|^2}{\omega_l - \omega_{ig}}\right) d_e + \sum_i \frac{\Omega^{(1)\star}_{ei}\Omega^{(1)\star}_{ig}}{\omega_l - \omega_{ig}} d_g. \tag{S6}$$

In the above equations, we identify frequency shifts $\Delta\omega_j = \sum_i \frac{|\Omega_{ij}|^2}{\omega_l - |\omega_{ij}|}$, $j \in \{e, g\}$ and the effective coupling constant between the ground and excited states

$$\Omega^{(2)} = \sum_i \frac{\Omega_{ig}^{(1)}\Omega_{ei}^{(1)}}{\omega_l - \omega_{ig}}. \tag{S7}$$

Note that this result can be also derived from the second order perturbation theory [4]. The effective Hamiltonian takes the form given in Eq. (1). This result justifies the two-level model assumed in *section: Semiclassical description of TPA*.

To account for the presence of the plasmonic nanostructure, each of the contributions of the effective coupling strength (S7) arising due to the presence of the intermediate states is rescaled by factors related to the plasmonic enhancement of the field component parallel to the corresponding electric dipole moment elements $\mathbf{d}_{ig}$ and $\mathbf{d}_{ei}$ of the transitions involving the virtual states:

$$\Omega_{\mathrm{NP}}^{(2)} = \sum_i \underbrace{\frac{\mathbf{E}(\omega_l, \mathbf{r}_m) \cdot \mathbf{d}_{ei}}{\mathbf{E}_0(\omega_l) \cdot \mathbf{d}_{ei}} \frac{\mathbf{E}(\omega_l, \mathbf{r}_m) \cdot \mathbf{d}_{ig}}{\mathbf{E}_0(\omega_l) \cdot \mathbf{d}_{ig}}}_{\text{enhancement factors}} \qquad \underbrace{\frac{\Omega_{ig}^{(1)} \Omega_{ei}^{(1)}}{\omega_l - \omega_{ig}}}_{\text{free-space two-photon coupling}} .$$

Assuming all transition dipoles to be co-oriented, the two-photon-transition coupling strength with the external field becomes rescaled by the field enhancement factors, as given in Eq. (2).

To verify the approximation, we solve the stationary master equations (3) from the main text with the full and effective Hamiltonians. We choose the parameters in the same range as those considered in the main text: $\omega_e = 2.7$ PHz, $\omega_i = 1.1$ PHz, $\gamma_{eg} = 20$ kHZ, $\gamma_{ig} = 20$ Hz, leading to $\delta_{ig} = 0.1\omega_{eg}$. The results are in excellent agreement, as shown in Fig. S1 for the excited state populations in the range of the light-matter coupling strengths $g_0 = \Omega_{ei}^{(1)} = \Omega_{ig}^{(1)}$ up to 100 MHz, as investigated in the main file.

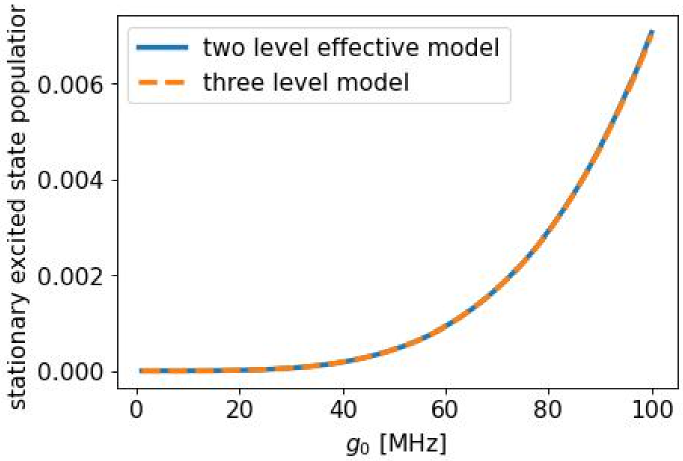

Figure S1: Comparison of the stationary excited-state population for modelled via the full Hamiltonian (orange dashed) and the effective one based on the adiabatic elimination of the intermediate state (blue solid line). The comparison is made for a range of single-photon coupling strengths $g_0 \equiv \Omega_{ei}^{(1)} = \Omega_{ig}^{(1)}$. The parameters are $\omega_e = 2.7$ PHz, $\omega_i = 1.1$ PHz, $\gamma_{eg} = 20$ kHZ, $\gamma_{ig} = 20$ Hz.

2 Plasmonic nanostructure without mirror film

In Fig. S2, we present the radiated power and electric field enhancement spectra for a nanostructure geometry without the bottom mirror film. A comparison with the structure

including the mirror film is presented in Fig. 5 of the main manuscript.

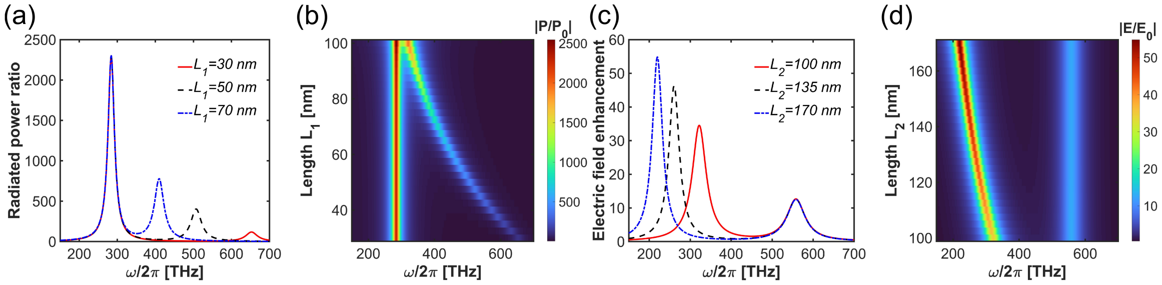

Figure S2: (a) Radiated power for selected lengths, and (b) radiated power spectrum by varying L_1 (c) Electric field enhancement, and (d) electric field enhancement spectrum by varying L_2.

References

[1] M. P. Fewell, "Adiabatic elimination, the rotating-wave approximation and two-photon transitions," *Optics communications*, vol. 253, no. 1-3, pp. 125–137, 2005.

[2] B. W. Shore, "Pre-history of the concepts underlying stimulated raman adiabatic passage (stirap)," *Acta Physica Slovaca*, vol. 63, 2013.

[3] J. Hou, K. Słowik, F. Lederer, and C. Rockstuhl, "Dissipation-driven entanglement between qubits mediated by plasmonic nanoantennas," *Physical Review B*, vol. 89, no. 23, p. 235413, 2014.

[4] C. Cohen-Tannoudji, B. Diu, and F. Laloë, *Quantum Mechanics*. No. t. 1 in A Wiley - Interscience publication, Wiley, 1977.
